# Host and Water Microbiota Are Differentially Linked to Potential Human Pathogen Accumulation in Oysters

**DOI:** 10.1128/aem.00318-23

**Published:** 2023-06-15

**Authors:** Rachel E. Diner, Amy Zimmer-Faust, Emily Cooksey, Sarah Allard, Sho M. Kodera, Emily Kunselman, Yash Garodia, Marc P. Verhougstraete, Andrew E. Allen, John Griffith, Jack A. Gilbert

**Affiliations:** a University of California, San Diego, Department of Pediatrics, La Jolla, California, USA; b University of California, San Diego, Scripps Institution of Oceanography, La Jolla, California, USA; c Southern California Coastal Water Research Project, Microbiology Group, Costa Mesa, California, USA; d Environment, Exposure Science and Risk Assessment Center, University of Arizona Mel and Enid Zuckerman College of Public Health, Tucson, Arizona, USA; e J. Craig Venter Institute, Environmental and Microbial Genomics Group, La Jolla, California, USA; The Pennsylvania State University

**Keywords:** Pacific oyster, *Vibrio parahaemolyticus*, *Vibrio vulnificus*, aquaculture, environmental microbiology, fecal organisms, oyster microbiome, shellfish

## Abstract

Oysters play an important role in coastal ecology and are a globally popular seafood source. However, their filter-feeding lifestyle enables coastal pathogens, toxins, and pollutants to accumulate in their tissues, potentially endangering human health. While pathogen concentrations in coastal waters are often linked to environmental conditions and runoff events, these do not always correlate with pathogen concentrations in oysters. Additional factors related to the microbial ecology of pathogenic bacteria and their relationship with oyster hosts likely play a role in accumulation but are poorly understood. In this study, we investigated whether microbial communities in water and oysters were linked to accumulation of Vibrio parahaemolyticus, Vibrio vulnificus, or fecal indicator bacteria. Site-specific environmental conditions significantly influenced microbial communities and potential pathogen concentrations in water. Oyster microbial communities, however, exhibited less variability in microbial community diversity and accumulation of target bacteria overall and were less impacted by environmental differences between sites. Instead, changes in specific microbial taxa in oyster and water samples, particularly in oyster digestive glands, were linked to elevated levels of potential pathogens. For example, increased levels of V. parahaemolyticus were associated with higher relative abundances of cyanobacteria, which could represent an environmental vector for *Vibrio* spp. transport, and with decreased relative abundance of *Mycoplasma* and other key members of the oyster digestive gland microbiota. These findings suggest that host and microbial factors, in addition to environmental variables, may influence pathogen accumulation in oysters.

**IMPORTANCE** Bacteria in the marine environment cause thousands of human illnesses annually. Bivalves are a popular seafood source and are important in coastal ecology, but their ability to concentrate pathogens from the water can cause human illness, threatening seafood safety and security. To predict and prevent disease, it is critical to understand what causes pathogenic bacteria to accumulate in bivalves. In this study, we examined how environmental factors and host and water microbial communities were linked to potential human pathogen accumulation in oysters. Oyster microbial communities were more stable than water communities, and both contained the highest concentrations of Vibrio parahaemolyticus at sites with warmer temperatures and lower salinities. High oyster V. parahaemolyticus concentrations corresponded with abundant cyanobacteria, a potential vector for transmission, and a decrease in potentially beneficial oyster microbes. Our study suggests that poorly understood factors, including host and water microbiota, likely play a role in pathogen distribution and pathogen transmission.

## INTRODUCTION

Bivalves are ecologically important animals that provide valuable coastal services and are a globally popular, potentially sustainable food source. In addition to recreational and subsistence harvesting, bivalve aquaculture operations generate numerous jobs and approximately US$23 billion in annual revenue (1). Oysters and mussels build coastal reefs which provide habitat for coastal organisms and can prevent erosion (2, 3). Additionally, adult bivalves filter large quantities of water to concentrate phytoplankton, which can reduce nutrient loads and improve coastal water quality (3).

The ability of bivalves to thrive in highly fluctuating coastal environments can be impacted by human activities and environmental stress, impairing the provision of these ecosystem services and their commercial potential (4). Additionally, due to their filter-feeding nature, many bivalves concentrate human pathogens, marine toxins, and coastal pollutants, which can endanger human health when consumed. These risks to seafood safety and security are likely to increase in the future due to global changes, such as climate change, increasing human populations, pollution discharge, and rapid coastal development (reviewed in references 5 and 6).

Bacterial pathogens are a major food safety concern, especially for oysters, which are commonly consumed raw (7). Infections can be caused by fecal-borne microbes from terrestrial sources and marine species that thrive in the oyster’s natural environment. For example, *Vibrio* species cause an estimated 80,000 human illnesses in the United States annually and 100 deaths (8). Among these, Vibrio parahaemolyticus is the most common cause of infection, while Vibrio vulnificus causes the greatest mortality, with 1 in 5 cases resulting in death. As these species thrive under warm conditions, they are highly concerning amid rising global seawater temperatures (9, 10). Additionally, since *Vibrio* spp. naturally occur in marine and brackish waters, they may resist depuration processes commonly employed to remove pathogens from oysters prior to entering the food supply. Understanding the factors that cause bacterial pathogens to accumulate in bivalves is critical to preserving human and ecosystem health now and in the future.

The environmental drivers of human pathogen concentrations in coastal waters are relatively well-characterized; however, connections between water and oyster pathogen concentrations are not fully understood. For fecal-borne bacteria, high concentrations in coastal waters are often linked to runoff from land-based sources and sewage spills, frequently coinciding with high concentrations of nutrients and other pollutants. Marine *Vibrio* species are associated with warm water temperatures and exhibit species-specific salinity distributions (reviewed in references 11 and 12). While the presence of pathogenic bacterial taxa in the water may be a prerequisite for oyster accumulation, studies investigating water-to-oyster pathogen transfer are either rare or yield inconsistent results, suggesting that factors beyond the environment likely play a role. For example, individual oysters from the same environment can have highly variable pathogenic bacterial concentrations; this is known as the “hot oyster” phenomenon, whereby certain oysters are more dangerous to consume than others because they accumulate more pathogens (13, 14). Ultimately, more research is needed to examine different variables that could explain the relationships between environmental factors, water-to-oyster human pathogen transfer, and pathogen accumulation and persistence in oysters.

Microbial communities associated with water and oysters are understudied variables that likely influence human pathogen accumulation. Microbial taxa in the water column may help pathogenic species proliferate in the environment and act as potential vectors for uptake and concentration by shellfish. For example, *Vibrio* species have been linked to phytoplankton concentrations in coastal waters, and human-pathogenic species have shown associations with specific phytoplankton groups and individual taxa that oysters may consume (15–19). Furthermore, environmental factors could influence or destabilize the oyster microbiome, impacting potential pathogen accumulation. Marine animal microbiomes often contribute to host health; critical functions include food breakdown, defense against host-associated pathogens, and modulation of host immune responses (reviewed in references 20 to 22). Under stressful conditions, however, animal microbiota can shift from a health-associated state to “dysbiosis,” whereby the microbiota and host no longer express beneficial synergy. This often leads to proportional increases in environmental microbes that are typically rare or absent in host microbiomes, or an increase in commensal host microbiota that can become pathogenic. Oyster pathogens such as the Ostreid herpesvirus 1 (OsHV-1), which is a growing concern in bays in California, can also contribute to microbiome dysbiosis (23). In aquaculture organisms, the accumulation of human pathogens in animal hosts as a function of dysbiosis has not been adequately investigated.

To investigate the influence of these understudied factors on potential human pathogen accumulation in oysters, we quantified fecal indicator bacteria (FIB), Vibrio parahaemolyticus and Vibrio vulnificus, across a natural environmental gradient in Newport Bay, CA, and characterized associated water and oyster microbiomes using metabarcoding. We then pursued the following objectives: (i) define relationships between water and oyster bacteria concentrations, (ii) determine how environmental variation influences oyster microbiomes and whether divergent microbiomes are linked to potential pathogen accumulation, and (iii) identify specific microbial taxa in water and oyster microbiomes that correlate with human pathogen accumulation in oysters, which could indicate environmental reservoirs or vectors for pathogen transmission.

## RESULTS

### Environmental and oyster collection conditions at experimental sites.

**FIG 1:**
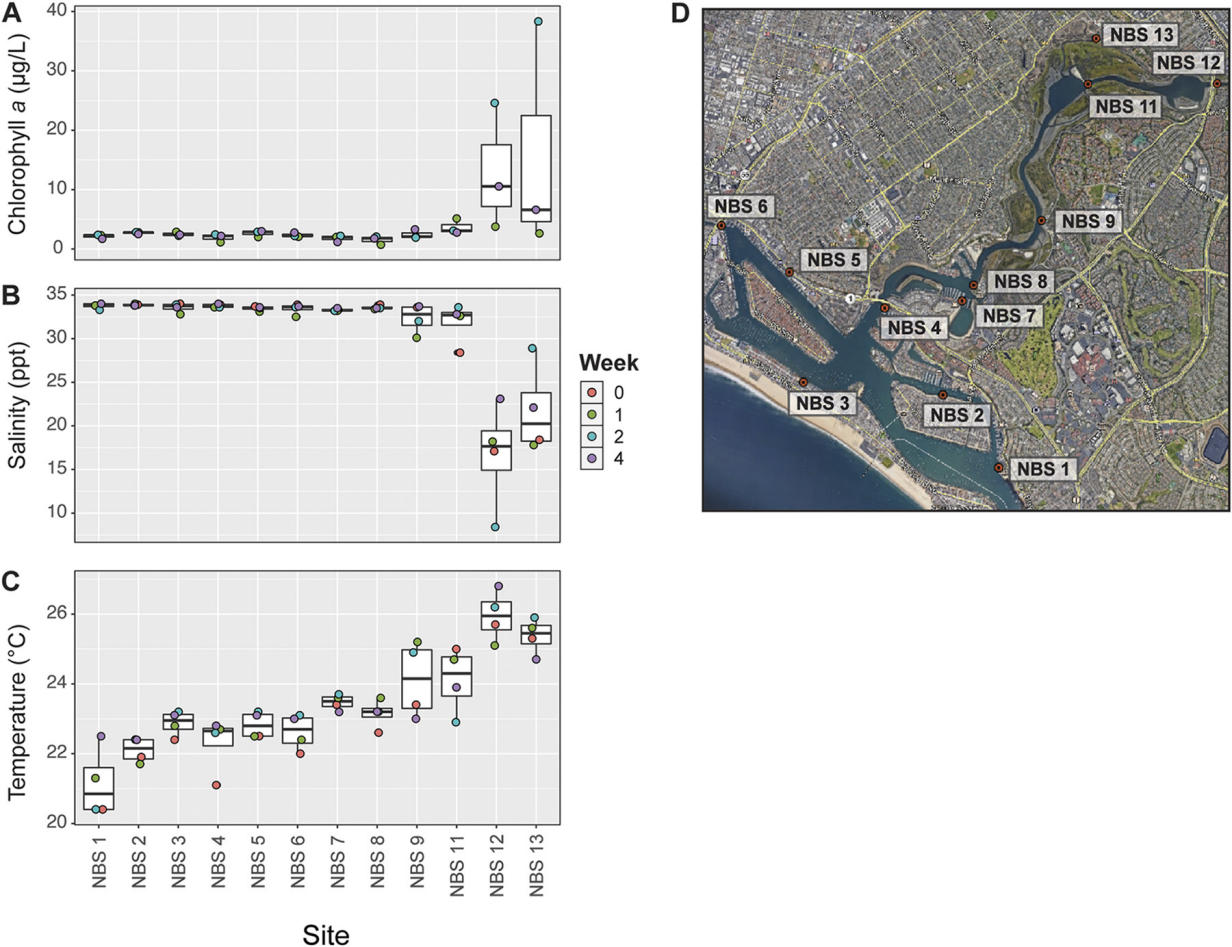
(A to C) Experimental sites and environmental conditions at the time of sample collection, including chlorophyll *a* concentration (A), salinity (B), and temperature (C). (D) Sites in Newport Bay, CA, where oysters were deployed and water and oyster samples were collected for the study. Map was generated using Google Earth with the following data attributions: Google, CSUMB SFML, CA OPC, USGS.

Experimental sites in Newport Bay, CA, differed in environmental conditions, including chlorophyll *a*, temperature, and salinity (Fig. 1A to D; see also Data Set S1 in the supplemental material). Chlorophyll *a* concentration ranged from 0.7 μg/liter to 38.3 μg/liter (Fig. 1A) and differed significantly by site (Kruskal-Wallis rank sum test, chi-squared = 23.164, *P* = 0.017). Sites close to the back of the bay (i.e., NBS12 and NBS13) exhibited the highest observed concentrations, which occurred during weeks 2 and 3 (Fig. 1A; Data Set S1). Temperature and salinity levels were also significantly different between sites (temperature: chi-squared = 29.261, *P* = 0.002; salinity: chi-squared = 23.598, *P* = 0.015). Salinity ranged from 8.4 to 34 ppt (Fig. 1B), and temperature ranged from 20.4 to 26.8°C (67.7 to 80.2°F) across all sites (Fig. 1C). Sites NBS12 and NBS13 exhibited warmer temperatures and lower salinities than other sites (Fig. 1B and C).

### Target bacteria species concentrations in seawater and oysters.

**FIG 2:**
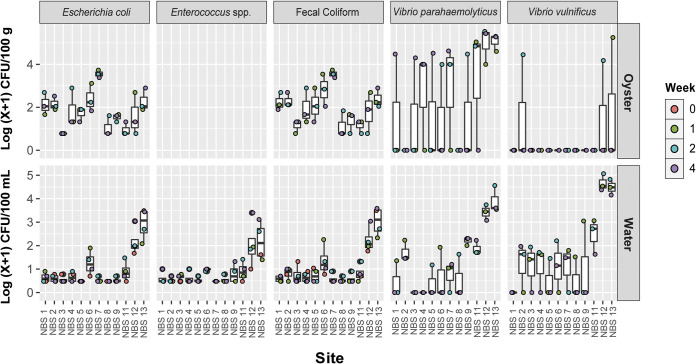
FIB and pathogenic *Vibrio* spp. concentrations in oysters and water. Concentrations are shown as log(x + 1) CFU/100 g of oyster tissue or 100 mL of seawater. FIB concentrations in oysters were calculated using the MPN method. *Enterococcus* bacteria were quantified in water but not in oyster samples.

FIB concentrations, a common proxy for human fecal contamination, varied by site and between water and whole oyster samples (termed “oyster samples” here) (Fig. 2; Fig. S1). Fecal coliform bacteria (FC), Escherichia coli, and *Enterococcus* spp. in water samples were elevated at sites 12 and 13 compared to samples collected at other sites during the same week (Fig. 2) (FC: chi-squared = 23.476, *P* = 0.015; E. coli: chi-squared = 28.760, *P* = 0.002). Oyster and water concentrations were not correlated for E. coli and fecal coliforms (linear regression, E. coli: F-statistic, 0.692769327, and adjusted *P* value, 0.411; fecal coliform: F-statistic, 1, adjusted *P* value: 0.191619216) (Fig. S1).

*Vibrio* species of concern for human health also varied in concentration by site, but unlike with FIB, oyster and water samples were positively correlated, particularly for Vibrio parahaemolyticus (Fig. 2; Fig. S1). In water, *Vibrio* spp. targets were highest at sites 12 and 13 compared to different sites measured during the same week (Fig. 2), which generally corresponded to warmer temperatures and lower salinities (Fig. 1B and C). Correlations between water and oyster concentrations were positive and significant for V. parahaemolyticus (Tobit regression, Wald-statistic = 5.862, *P* = 0.015) and for V. vulnificus (Tobit regression, Wald statistic = 5.917, *P* = 0.015), though for V. vulnificus only 3 data points had positive values for oyster and water concentrations, and thus further validation is needed (Fig. S1).

### Influence of sample type on microbial community diversity and composition.

We characterized microbial communities using 16S rRNA V4-5 amplicon sequencing for 4 different sample types: water, whole oysters, oyster gill tissue, and oyster digestive glands (DG). After filtering for quality, ~11 M sequences were generated across samples and controls, representing ~33,000 unique amplicon sequence variants (ASVs) belonging predominantly to bacteria.

**FIG 3:**
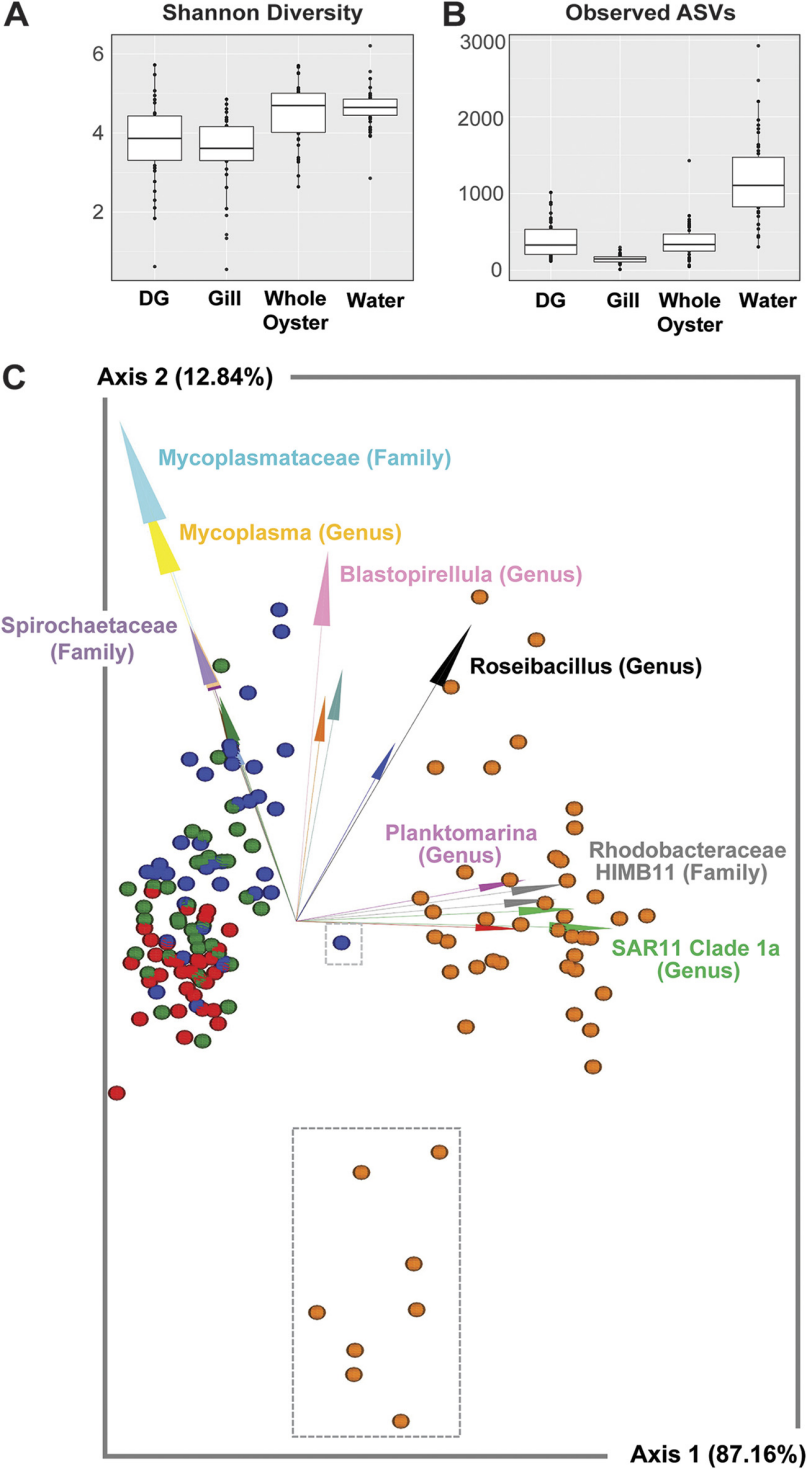
Diversity of water and oyster microbial communities and relationships with environmental parameters and pathogen concentrations. (A and B) Shannon diversity (A) and observed ASVs (B) compared between sample types. (C) DEICODE biplot showing RPCA distances between samples colored by sample type (blue, digestive gland; green, whole oyster; red, gill; orange, water) and arrows depicting the key microbial taxa driving diversity, with annotation to the highest available taxonomic annotation noted. Water samples from sites NBS12 and NBS13 are enclosed in the larger gray dotted box, and a DG sample from NBS12 at time point 2 is enclosed in the smaller gray box.

Sample type was a major driver of microbial diversity, with significant differences in both alpha and beta diversity. Water samples had higher species richness (observed ASVs) than all oyster sample types, and water and whole oysters had higher alpha diversity (Shannon diversity) than the oyster digestive gland and gill tissue (Fig. 3A and B; Data Set S2). Sample type significantly influenced beta diversity (permutational multivariate analysis of variance [PERMANOVA]: pseudo-F = 122.014, *P* = 0.001) (Data Set S2), and sample types significantly differed from each other based on pairwise PERMANOVA tests (robust Atchison principal-component analysis [RPCA]) (Fig. 3C; Data Set S2).

Specific microbial taxa were strongly associated with either oyster or water sample types, or in some cases, specific oyster tissues. Key microbial features characteristic of oyster samples included members of the *Mycoplasmataceae* (class *Bacilli*) and *Spirochaetaceae* families (class *Spirochaetia*) (Fig. 3C), which were also among the most relatively abundant taxa found in oyster tissues generally (Fig. S2, Data Set S3). Key water taxa included marine members of SAR11 Clade Ia, HIMB11, and *Planktomarina* (Fig. 3C), which are all in the class *Alphaproteobacteria*. While oyster gill and DG microbiomes shared some common microbial taxa, others were more representative of either sample type. For example, members of the genus *Blastopirellula* (class *Planctomycetes*) and genus *Spiroplasma* (class *Bacilli*) were more representative of DG samples, while *Sphingoaurantiacus* (class *Alphaproteobacteria*) and *Spirochaetia* ASVs had high relative abundance in gill but not DG samples (Fig. S2).

### Influence of site and environment on microbial community diversity and composition.

Water microbial community diversity was impacted by site and environmental conditions to a greater extent than were oyster microbiomes, which were comparably stable across sites. Beta diversity of water samples differed at back bay sites, which also exhibited differences in temperature, salinity, and chlorophyll *a* concentration (Fig. 1). Communities from NBS12 and NBS13 were distinct from other sites, as indicated by the gray dashed box surrounding these samples in the biplot (Fig. 3C) and in pairwise distance comparisons between sites within water samples (Data Set S4). Alpha diversity (observed ASVs and Shannon diversity) was negatively associated with temperature and positively associated with salinity in water samples, suggesting lower diversity at bay back sites (Fig. S3).

Beta diversity in oyster samples did not significantly differ across sites and environmental conditions, but there were site-specific differences in taxonomic composition and environmentally linked differences in tissue-specific alpha diversity. Water and DG samples exhibited high relative abundance of a cyanobacterial ASV annotated as *Cyanobium* PCC-6307, which is commonly found in freshwater aquatic environments (Fig. 2). Chlorophyll *a* levels were high during these sampling points, which may suggest high actual and not just relative abundance of these cyanobacteria. Additionally, gill alpha diversity was associated with warmer temperatures (Fig. S3).

**FIG 4:**
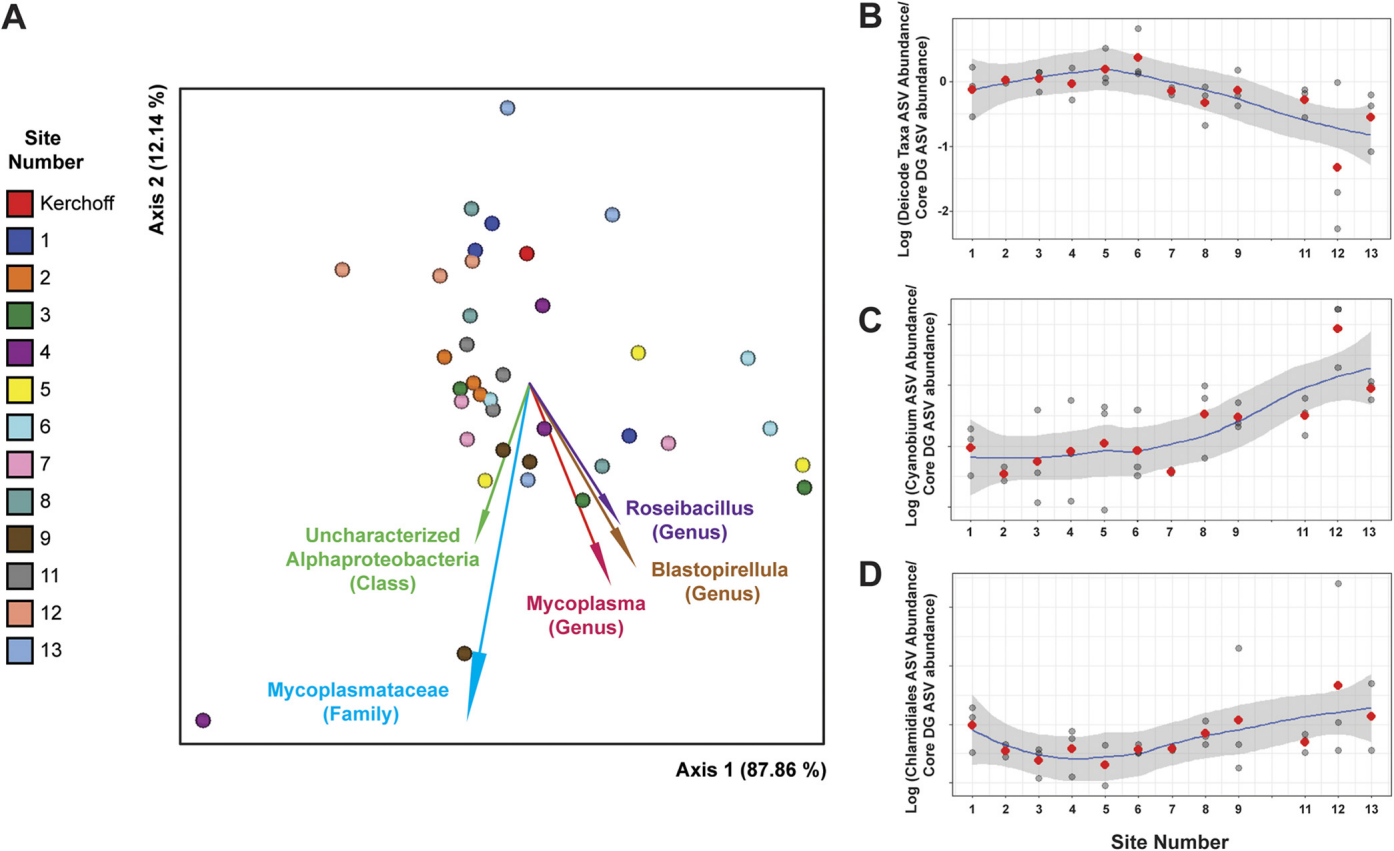
Differentially abundant taxa across sites in oyster digestive gland tissues. (A) RPCA biplot of digestive gland samples with markers depicting individual samples and marker colors indicating site (shown in legend). Arrows depict microbial taxonomic features driving differences in community composition (beta diversity) with the best available taxonomic composition. (B ti=o D) The log fold change in differential abundances (compared as a ratio to the core digestive gland microbiome) is shown for the driving taxonomic features from the RPCA DEICODE analysis (taxa with arrows) (B) and two specific ASVs common at back bay sites: *Cyanobium* PCC-6307 (C), and a *Chlamydiales* ASV (D). Red markers denote the average log fold changes.

Additional differences in oyster microbial community taxonomy were observed at NBS12. Key taxa associated with DG microbiomes (Fig. 4A, taxa annotated with arrows) had lower relative abundance at this site than did the core DG microbiome (Fig. 4B). Additionally, during week 2 in DG samples, a *Chlamydiales* ASV comprised ~30% of the microbial community. These are often intracellular animal pathogens and have been hypothesized to be linked to oyster edema disease in pearl oysters (24). Both the *Cyanobium* and *Chlamydiales* ASVs had higher differential abundance in relation to the core DG microbiome at NBS12 compared to other sites (Fig. 4C and D). The increase of these two ASVs at this time point also manifested as a community with distinct beta diversity (this data point is enclosed in a gray dotted box in the biplot in Fig. 3C); however, this could not be statistically tested, as oysters in this sample were pooled.

### Relationship between potential bacterial pathogens and water or oyster microbial communities.

We investigated whether certain characteristics of water and oyster microbial communities, namely, community diversity and prominent microbial taxa in these sample types, were associated with high or low concentrations of fecal indicators and target *Vibrio* species. In water samples, microbial communities with higher fecal indicator and *Vibrio* spp. levels were typically found at the back bay sites NBS12 and NBS13 and were negatively correlated with salinity and ASVs common in marine environments (e.g., SAR11 Clade Ia, HIMB11, and *Planktomarina*) (Fig. 2 and 3; Fig. S4). Some specific microbial taxa were linked to low-salinity conditions found at back bay sites and high potential pathogen concentrations (Fig. S4). For example, a *Flavobacterium* ASV was positively associated with multiple bacteria of concern, including elevated Vibrio parahaemolyticus concentrations in both water and oysters. Additionally, *Cyanobium* PCC-6307 coincided with elevated V. parahaemolyticus levels in water, particularly at site NBS12 during weeks 2 and 4.

In oyster samples, beta diversity was more stable across sites than in water; however, several specific microbial taxa were associated with either fecal indicator or *Vibrio* spp. concentrations. In whole oyster samples, *Mycoplasma* and *Blastopirellula* ASVs were negatively correlated with concentrations of bacterial targets in water (Fig. S5). We also separately assessed gill and DG microbiomes. In DG microbiomes, ASVs belonging to the *Blastopirellula* and *Mycoplasma* genera were negatively associated with V. parahaemolyticus in oysters, which likely drove the trend observed in whole oysters. Additionally, *Fuertia* bacteria (order *Planktomycetales*) were associated with lower levels of E. coli and fecal indicator bacteria. In gill samples, most of the dominant taxa besides *Mycoplasma* ASVs were not significantly associated with environmental variables or human health-associated bacteria (Fig. S7). The cyanobacteria taxon *Cyanobium* PCC-6307 had high relative abundance in water and oyster DG microbial communities containing high concentrations of V. parahaemolyticus (site NBS12, weeks 2 and 4) and was positively associated with V. parahaemolyticus concentrations in oysters (Fig. S6).

In addition to examining correlations between pathogen concentrations and relative abundance of microbial taxa, we used a differential abundance approach to assess whether other microbial taxa common in DG or water samples decreased in relation to other taxa (i.e., a compositionally aware approach rather than solely examining relative abundance). This addressed whether the observed decrease in relative abundance of key taxa was due solely to the proportional increase in *Cyanobium* PCC-6307 or whether ratios of these taxa to the core DG microbiome also increased. While the log ratio of *Cyanobium* to core DG taxa increased at site NBS12, the ratio of several key taxa of interest (as indicated by the DEICODE RPCA analysis and biplot [Fig. 4A]) decreased in relative abundance, suggesting a decrease in the abundance of these taxa.

## DISCUSSION

### Oyster and water microbiota are differentially influenced by the environment.

Understanding how the environment influences oyster microbiomes is critical to preserving their ecosystem functions and supporting safe and abundant shellfish harvesting. Our study focused on whole oyster as well as gill and DG microbial communities. In line with previous studies (25, 26), all sample types we examined had significantly distinct microbial communities (Fig. 3C; Fig. S2) and differed from the surrounding water communities. The dominant tissue-specific taxa we observed were consistent across sites and consistent with taxa common in Pacific oysters from diverse geographic regions, suggesting these taxa may be adapted to host microenvironments.

*Spirochaetaceae* taxa, identified in all 3 sample types in various proportions, have been previously observed in *Crassostrea gigas* gill samples (25–28). Members of this family can cause human disease (e.g., Lyme disease and syphilis) but are also commonly associated with marine animals ranging from gastropods to corals; they provide beneficial host functions, including nitrogen and carbon fixation (29–32). *Mycoplasmataceae* bacteria were common in DG samples, a pattern observed in the above studies and in samples from France (33) and in Olympia oysters (*Ostrea lurida*) from the northwest United States (34). Interestingly, this was not a dominant DG microbial group in Pacific oyster DG samples from Mexico; however, this may have been due to different analytical methods (35). Like *Spirochaetaceae*, some *Mycoplasmataceae* taxa are implicated in human and animal disease, while others naturally reside in animal microbiomes with potentially symbiotic roles. A metagenomic study of Eastern oyster (*Crassostrea virginicus*) microbiomes identified a dominant *Mycoplasma* sp. with a reduced set of metabolic functions and high reliance on host-derived nutrients (36), suggesting longstanding symbiosis. For these taxonomic groups, their role in oysters has not been characterized in depth; however, it is possible that changes in the relative abundance of these taxa due to environmental or other stressors could have negative host impacts.

As our experimental sites represent a range of environmental conditions and oyster microbiomes can shift under stress (22, 37, 38), we predicted that microbial communities in both water and oysters would differ across sites. Despite distinct water microbiomes at each site, oyster microbial communities remained consistent (Fig. 3C; Data Set S4). This may reflect a strong selection pressure for the structure of these communities. It is also possible that short-term (i.e., weeks) exposure to these conditions was not sufficient to impact community diversity. Some differences in oyster microbiomes were observed for the 2 sites with significantly lower salinity and higher temperature. These sites and environmental differences were linked to a lower relative abundance of *Mycoplasmataceae* taxa in DG tissues, which could reflect a detrimental impact to hosts if these bacteria are beneficial.

### Potential human pathogen concentrations were linked to specific environments and microbial community features.

Several factors likely play a role in bivalve pathogen accumulation, including environmental pathogen concentrations, abundance and distribution of microbial virulence genes, host behavior (e.g., feeding), pathogen interactions with bivalve transmission vectors, host physiology (including microbiome states), and microbial features that enable host colonization. Our study investigated these latter microbial variables, which are a key and understudied factor in human pathogen ecology in the marine environment and could in the future lead to better prediction of bivalve pathogen concentrations. We examined potential microbial pathogens at the species or genus level; however, future studies should integrate the presence of known virulence genes into this framework, as not all species members can actually cause disease.

We predicted that oysters translocated to potentially stressful locations (i.e., sites NBS12 and NBS13) would concentrate more potential human pathogens, with coinciding microbial community changes. This occurred in the case of Vibrio parahaemolyticus, the most common cause of seafood transmitted vibriosis disease (4), but it did not occur with regard to V. vulnificus or fecal indicators measured, which exhibited variable patterns across environmental sites and over time. In water, FIB and *Vibrio* spp. bacteria were most abundant at sites NBS12 and NBS13, consistent with their affinities for low salinity and warm temperatures. Fecal indicator and *Vibrio* spp. concentrations in oysters, however, were generally not linked to water concentrations or environmental conditions (except for V. parahaemolyticus, discussed below), though in the case of V. vulnificus the low number of detections could confound the correlation analysis. Oysters with stable microbial communities could potentially accumulate environmental human pathogens through feeding and filtering but possess microbiome-regulated controls on total accumulation (e.g., microbial competition for nutrients or space within tissues). This could explain why oysters in water that contained relatively high V. vulnificus or FIB concentrations (i.e., the back bay sites NBS12 and NBS13) did not accumulate higher concentrations of these organisms than oysters at other sites. This also confirmed that while environmental conditions conducive to pathogen proliferation may be necessary for high concentrations in water, they are not sufficient for accumulation in shellfish.

In the case of V. parahaemolyticus, concentrations in water and oyster samples were positively correlated and linked to changes in oyster microbial communities and environmental conditions. The highest oyster V. parahaemolyticus concentrations coincided with a distinct DG microbial community (Fig. 3C, small gray dotted box) and an increase in the relative abundance of two uncommon DG taxa, a Chlamydiae family ASV and a cyanobacteria taxon *Cyanobium* PCC-6307 (Fig. 4C and D). Meanwhile, several common DG oyster taxa (e.g., *Mycoplasma* and *Blastopirellula*) were less relatively abundant at the “high V. parahaemolyticus” back bay sites. Since *Mycoplasma* bacteria are considered core members of oyster DG microbial communities and have been linked to increased oyster survival (34), the increase in rare taxa and decrease in core taxa may suggest poor host health and microbial community dysbiosis. V. parahaemolyticus was also consistently abundant at site 13, which had similar environmental conditions to NBS12 but not these divergent taxa. This suggests that both the environment and specific microbial taxa in oysters act synergistically to enable marine human pathogen accumulation in oysters. Other factors may also be involved, including time oysters are exposed to stressful environments and oyster feeding behavior.

In investigating these associations between oyster microbiomes and fecal indicator and *Vibrio* spp. concentrations, some possible confounding variables in this data set include the possibility that oyster microbiomes may change over time following translocation and that pathogens may take time to accumulate in oyster tissues. Specific properties of the sites where oysters are collected could influence oyster microbiomes after pooling, depuration, and deployment, and it is unknown how rapidly the oyster microbiomes stabilize and adapt to new conditions. Additionally, pooling multiple oyster samples, which is a common methodology for quantifying human pathogens, obscures variability of individual oysters in samples. Finally, the sites included in this study had varied physiochemical profiles. Although this allowed us to explore the effects of salinity and temperature, additional factors that were not measured may have differed between sites and could have influenced the results as well. These factors present some limitations to the current experiment that should be considered.

### Phytoplankton links to oyster pathogen accumulation.

The highest V. parahaemolyticus concentrations observed in oysters were positively correlated with high relative abundance of cyanobacteria in water and oyster DG samples and high water chlorophyll *a* concentrations (Fig. 1 and 2, Fig. S2, Fig. S6). This led us to consider whether and how phytoplankton might influence the transport of V. parahaemolyticus into oysters. Phytoplankton could potentially stimulate uptake of V. parahaemolyticus from the environment if perceived as a food source by oysters. Furthermore, it is possible that V. parahaemolyticus attached to cyanobacteria or other phytoplankton (eukaryotic algae were not assessed in this study), which could enhance oyster V. parahaemolyticus accumulation. V. parahaemolyticus has been shown to interact with cyanobacteria and attach to diatom phytoplankton taxa in the laboratory (19, 39). Additionally, we previously observed several phytoplankton taxa that were positively linked to high V. parahaemolyticus concentrations in southern California water microbiomes, including species that oysters feed on (17). We did not examine actual V. parahaemolyticus attachment to phytoplankton or oyster feeding behavior in this study, but this potential mechanism for enhanced V. parahaemolyticus uptake should be explored mechanistically in future studies.

Alternatively, cyanobacteria could be indirectly or unrelated to the observed V. parahaemolyticus concentrations. If cyanobacteria were not consumed as food by oysters, they could instead be an indication of stress-induced microbiome dysbiosis (discussed above). This could also influence the prevalence of environmental bacteria such as V. parahaemolyticus. Finally, it is possible that environmental conditions drive both V. parahaemolyticus concentrations and the relative abundance of cyanobacteria in water and oysters but that the two microbial populations are not interacting or related to each other and their cooccurrence is coincidental. Integrating knowledge of the oyster microbiome with host behavior and pathogen microbial ecology will help elucidate the interactions between both host- and microbe-mediated drivers of bivalve pathogen accumulation, particularly in relation to predicted future ocean changes.

### Conclusion.

Understanding factors beyond environmental conditions that drive human pathogen accumulation in bivalves is critical to supporting safe and sustainable seafood consumption. Here, we demonstrate that both environmental factors and microbial communities interact to differentially influence concentrations of potential human pathogens in water and oysters. While specific environmental conditions are linked to both microbial community diversity and concentrations of potential pathogenic *Vibrio* spp. and indicator species of fecal contamination in water samples, concentrations of these bacteria in water are not necessarily linked to concentrations in oysters. Oyster microbiomes and pathogen concentrations were less environmentally dependent than those in water, except in the case of Vibrio parahaemolyticus, where relatively high oyster concentrations were associated with an increase in environmental V. parahaemolyticus and cyanobacteria and a decrease in the relative abundance of key digestive gland taxa. This study suggests that environmental conditions and microbial communities may interact, potentially synergistically, to drive human pathogen concentrations in oysters. Future research integrating pathogen attachment to oyster uptake vectors in the environment, oyster behavior and physiology, and the functional roles of oyster microbiomes and specific taxa, particularly in response to changing environmental conditions, will provide data critical for promoting safe seafood harvesting for a growing human population in the future.

## MATERIALS AND METHODS

### Experimental design and sample collection.

Approximately 1,200 Pacific oysters were collected over a 3-day period (31 July to 2 August 2019) from 3 sites across Newport Bay, CA, and then transported to holding tanks located at the Kerckhoff Marine Laboratory in Corona Del Mar, CA, within 4 h of harvesting (40). At the Kerckhoff Marine Laboratory, oysters were pooled and arranged on perforated stacked trays in four 282.7 m^3^ flowthrough seawater tanks for 14 days. This “depuration” procedure is a standard treatment for harvested bivalves aimed at reducing the number of bacterial pathogens from fecal and terrestrial sources. Seawater was first filtered through a sand filter at 15 to 20 gal per min and then further disinfected with a classic UV 80-watt series light (Aqua UV, Temecula, CA, USA) before entering the holding tanks.

Following the 2-week hold time,10 to 12 oysters were collected and processed from each holding tank to characterize composite postdepuration microbial communities in whole oysters (*N* = 10 to 12) and oyster tissue samples (gills and digestive glands [DG], *N* = 10 to 12 each). This was designated week 0 for oyster samples. The depurated oysters were then pooled and deployed across 12 sites in Newport Bay, CA (Fig. 1) (40), none of which were the original collection sites. Roughly 100 oysters were deployed in 23-mm plastic mesh oyster bags at each site. At the time of oyster deployment, ambient water samples were taken from each of the 12 sites (designated week 0 for water samples). Thereafter, both water and oyster samples were collected at weeks 1, 2, and 4. Grab water samples were collected from each site in cleaned and acid-rinsed (10% HCl) 2-liter polycarbonate bottles and held on ice until processing, while oyster samples were collected as composite samples of 10 to 12 individual oysters from each site at each time point and for each tissue type. Oysters were stored in coolers with ice packs and transported to the Southern California Coastal Water Research Project (SCCWRP) for further processing. Environmental data were collected using a YSI30 Pro field instrument (Yellow Springs, OH), which was used primarily to assess temperature and salinity.

Following depuration, oysters were translocated to identical cages and secured to each site via dock, mudflat, or seawall (Data Set S1). Oysters are endemic to all of the sample sites, with the exception of NBS12 and NBS13, where we did not observe oysters during field visits or during collection or deployment. The environmental variations observed at these sites (Fig. 1A to C) suggest that environmental or biotic factors may have made these sites inhospitable to endemic oyster growth; however, low oyster populations could also result from illegal harvesting.

### Sample processing and storage.

Approximately 100 to 200 mL of water was filtered onto 0.4-μm polycarbonate filters for nucleic acid extraction, which was used downstream to characterize water microbial communities. This pore size was selected to optimize bacterial pathogen quantification without clogging filters; however, as a result, microbial community members of <0.4 μm were not assessed in this study. Filtered samples were stored in 1.5-mL tubes and frozen in liquid nitrogen, followed by immediate storage at −80°C until DNA extraction. Water column chlorophyll *a* concentration was measured by filtration of 100 mL on 25-mm glass fiber filters (Whatman) with gentle filtration, with filters stored in aluminum foil packets at −80°C until processing. Filters for chlorophyll a were extracted in 100% acetone in the dark for 24 h. Total chlorophyll a was measured in all water samples using a nonacidification method and a Trilogy Turner Design fluorometer, as previously described (41). Additional water samples were processed according to standard methods for quantification of FIB, including Escherichia coli, total coliform bacteria, fecal coliform bacteria, and *Enterococcus* species (see below).

At SCCWRP, ~10 oysters were homogenized, and the composite was processed to quantify bacterial targets according to published methods (see below and Table 1). An additional ~10 oysters were shucked and dissected, and the gill and digestive gland tissues were separately pooled and homogenized. Homogenized digestive glands and gills were stored for downstream DNA extraction at −80°C.

**TABLE 1:** Molecular quantification methods for fecal indicator bacteria and target *Vibrio* spp.

Target bacteria	Matrix	Method^*a*^	Method reference	Gene target	PCR reference
Fecal coliform/Escherichia coli	Oyster	5-tube, 3-dilution MPN MTF (LST, EC-mug for confirmation)	APHA, 1970 (42)		
Fecal coliform/E. coli	Water	EPA method 1603: membrane filtration with mTEC	USEPA, 2009 (43)		
*Enterococcus* spp.	Water	EPA method 1600: membrane filtration on mEI	USEPA, 2009 (44)		
Vibrio parahaemolyticus	Oyster/Water	Spread plate on CHROMagar Vibrio + isolate confirmation by PCR	Froelich et al., 2017 (45)	*toxR*	Taiwo et al. 2017 (46)
Vibrio vulnificus	Oyster/Water	Spread plate on CHROMagar Vibrio + isolate confirmation by PCR	Froelich et al., 2017 (45)	*vvhA*	Warner and Oliver, 2008 (47)

a MTF, Multiple Tube Fermentation; mTEC, membrane-Thermotolerant Escherichia coli agar; mEI, membrane-Enterococcus Indoxyl-ß-D-Glucoside Agar.

### Quantification and detection of human pathogen and indicator species and OsHV-1 virus.

The bacterial targets quantified in this study and the associated analysis methods are listed in Table 1. Escherichia coli and fecal coliforms were quantified in oyster tissues following approved methods developed by the FDA and Interstate Shellfish Sanitation Conference (Table 1). *Enterococcus* bacteria were quantified in water but not in oyster samples. Briefly, fecal coliform and E. coli concentrations in oysters were determined by the conventional five-tube multiple dilution most-probable number (MPN) procedure. Lauryl tryptose broth (Difco) was utilized for the presumptive growth medium, with confirmation performed by inoculating liquid EC-MUG media (Difco) at 44.5°C for 2 ± 2 h. Grab water samples were processed for cultivable *Enterococcus*, E. coli, and fecal coliform CFU according to standard methods (EPA method 1600, EPA method 1603, and SM 9222-D) (42–44).

For *Vibrio* spp. targets, V. parahaemolyticus and V. vulnificus were quantified using a culture-based MPN method. Briefly, CHROMagar *Vibrio* (CHROMagar, Paris, France) media plates were prepared according to manufacturer’s instructions and used to enumerate potentially pathogenic *Vibrio* species. V. parahaemolyticus and V. vulnificus concentrations were determined by counting visible pink and blue colonies on the CHROMagar *Vibrio* medium, respectively, and adjusting for dilution (45). Data were reported as CFU per 100 mL and CFU per 100 g for water and oysters, respectively, and the limit of detection was 1 CFU/g or 1 CFU/mL. Up to 10 presumptive V. parahaemolyticus and V. vulnificus colonies per plate (when present) were stored for further species-level PCR confirmation (45–47). The number reported was then multiplied by the percentage of molecularly confirmed (by PCR) isolates, resulting in confirmed bacterial abundance for each sample, as described previously (48).

To test for OsHV-1 presence and abundance, the ORF100 primer set described by Burge et al. was used for quantitative PCR (49). Briefly, 10 μL of PerfeCTa SYBR green FastMix (Quantabio), 1 μL of each primer (ORF100 F, ORF100 R), 6 μL of water, and 2 μL of DNA template were mixed per reaction mixture. All samples were run in duplicate on the 96-well Agilent ARIAMx RT-PCR thermal cycler. A standard curve was generated with a synthetic plasmid of the ORF100 DNA sequence by serial dilution from 30 million copies down to 3 copies.

### Statistical analysis of environmental and target bacteria variables.

To determine whether the deployment site had a significant effect on concentrations of target bacteria and environmental conditions, nonparametric Kruskal-Wallis tests were conducted in R using the stats package. Pairwise comparisons were not conducted due to low sample number. Linear regression analyses were utilized to determine whether significant correlations existed between water and oyster samples for target bacteria. When data were not normally distributed as determined by the Shapiro-Wilk normality test, which occurred with V. parahaemolyticus and V. vulnificus, Tobit regression analysis was utilized to test for a significant relationship between variables.

### DNA extraction, amplicon library preparation, and sequencing.

DNA was extracted from water samples using the Qiagen PowerSoil kit and from oyster tissues using the Qiagen DNEasy blood and tissue kit (Qiagen, Hilden, Germany). Digestive gland and gill composites, which were homogenized at the time of collection, were digested with Proteinase K prior to DNA extraction. The V4-V5 region of the 16S rRNA gene was amplified using 515F-926R primers (50). Libraries were assessed for quality using an Agilent 2200 TapeStation (Agilent, Santa Clara, CA). Since these primers also produce amplicons from 18S rRNA, BluePippin size selection was used to enrich for ~550 bp amplicons prior to sequencing (Sage Science, Beverly, MA, USA). Additionally, 3 blanks (lysis buffer only, taken through extraction protocol, 2 extracted with tissue samples, and 1 run with water samples) and 2 samples of DNA from mock bacterial communities (ZymoBIOMICS Microbial Community DNA Standard, Zymo Research, Irvine CA) were prepared and sequenced, with all samples included in the same sequencing run. After library construction, samples were sequenced using the Illumina MiSeq 2 × 300 kit (v. 3) with custom adapters and dual barcode indices at the University of California Davis Genome Center (https://genomecenter.ucdavis.edu/).

### Sequence quality filtering and bioinformatic analysis.

The QIIME2 pipeline was used for quality control, filtering, and bioinformatic analysis (51). R packages, including Phyloseq (52), were used for additional analyses and data visualization. Demultiplexed sequences were imported as QIIME2 artifacts, and paired reads were trimmed to remove primers, merged, and assigned ASVs using dada2 (default parameters) (53). Taxonomy was assigned to ASVs using the SILVA database (version 138) (54). Prior to downstream analyses, mitochondrial, chloroplast, and eukaryotic sequences were removed as well as ASVs with no identified domain.

Diversity analyses were conducted in phyloseq without rarefaction, though rarefaction at a sampling depth of 2,298 (determined by alpha rarefaction curves) was also compared to confirm that results were consistent between rarefied and nonrarefied data sets. Pairwise comparisons of alpha diversity were conducted in R using a Wilcoxon rank sum test, using the Holm method for *P* value adjustment. For beta diversity analyses, RPCAs were conducted and then visualized as biplots using the QIIME2 DEICODE plugin (55), with a minimum feature count of 10 and a minimum sample count of 500. PERMANOVA tests were used to analyze pairwise differences between groups (e.g., sample type, site) using the qiime diversity beta-group-significance command.

Log fold changes in the differential abundance of key taxonomic features in digestive gland samples were conducted in R by calculating the log ratio of targeted taxa of interest to core digestive gland microbial taxa in individual samples. Microbial taxa of interest were identified using three approaches (identification via DEICODE biplots, identification via taxonomy plots, and identification of ASVs belonging to *Cyanobacteria*) and analyzed separately. Core microbes were identified using the qiime feature-table core-features command, which identified 10 taxa present in >85% of samples. One of these taxa, one *Serratia* ASV, was removed from core microbiome consideration as it was also found in blank samples and is not considered a marine or oyster-associated bacterial taxa. For each sample, the abundances of taxa of interest were compared against abundances of core microbial taxa to provide differential abundance values. We used the following formula:

$$\ln\left(\frac{\sum_{n=1}^{10}\text{Taxa of interest ASV abundance}}{\sum_{n=1}^{10}\text{Core of microbiome taxa ASV abundance}}\right)$$

Correlations between metadata variables, including alpha diversity metrics, environmental variables, and pathogen concentrations, were calculated based on Spearman’s rank correlations and visualized using the corrplot package in R (56). Additional correlations between these factors and the relative abundance of taxa of interest were conducted using data from weeks 0, 1, 2, and 4 for water samples and weeks 1, 2, and 4 from oyster samples. Samples collected at the Kerchoff facility following depuration (week 0) were not included in the correlation analyses.

### Data availability.

Raw sequence files and metadata files have been deposited with NCBI (BioProject PRJNA925853) and are also available on the Qiita platform (57) (study ID 14776).
