# Supplementary material for: Host and Water Microbiota Are Differentially Linked to Potential Human Pathogen Accumulation in Oysters
Source: Appl Environ Microbiol. 2023 Jun 15;89(7):e00318-23. doi: 10.1128/aem.00318-23 (PMC10370324; doi:10.1128/aem.00318-23)
Supplement: Supplemental file 1 — Supplemental material. Download aem.00318-23-s0001.pdf, PDF file, 0.7 MB [file aem.00318-23-s0001.pdf]

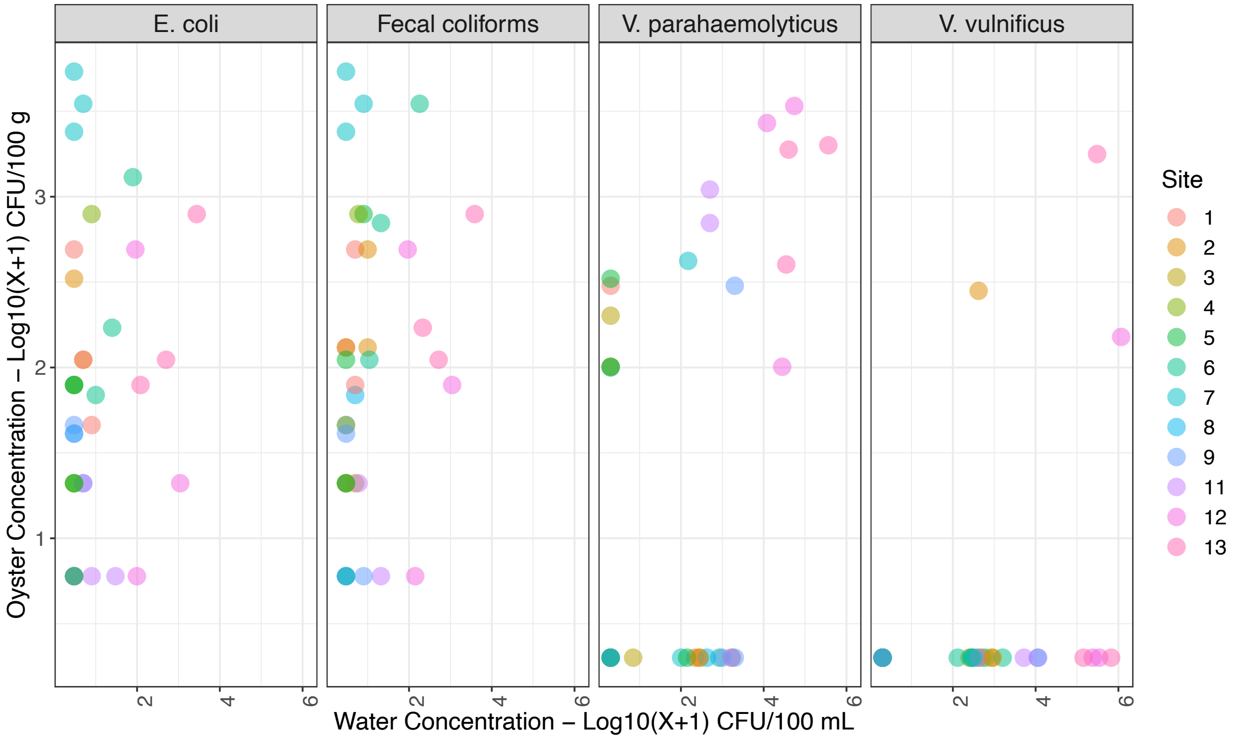

Fig S1: Relationships between water and oyster concentrations of fecal indicator bacteria (FIB) and target *Vibrio* species. FIBs include *Escherichia coli* and fecal coliform bacteria. *Vibrio* spp. targets include *V. parahaemolyticus* and *V. vulnificus*. Correlations between water and oyster concentrations were not significant for *E. coli* and fecal coliforms (Linear Regression, *E. coli*: F-statistic: 0.6927, adjusted p-value: 0.411, fecal coliform: F-statistic: 1, adjusted p-value: 0.1916). Correlations between water and oyster concentrations were significant for *V. parahaemolyticus* (Tobit Regression, Wald-statistic: 5.862, p-value: 0.015) and for *V. vulnificus* (Tobit Regression, Wald-statistic: 5.917, p-value: 0.015), though for *V. vulnificus* only 3 datapoint had positive values for both oyster and water concentrations.

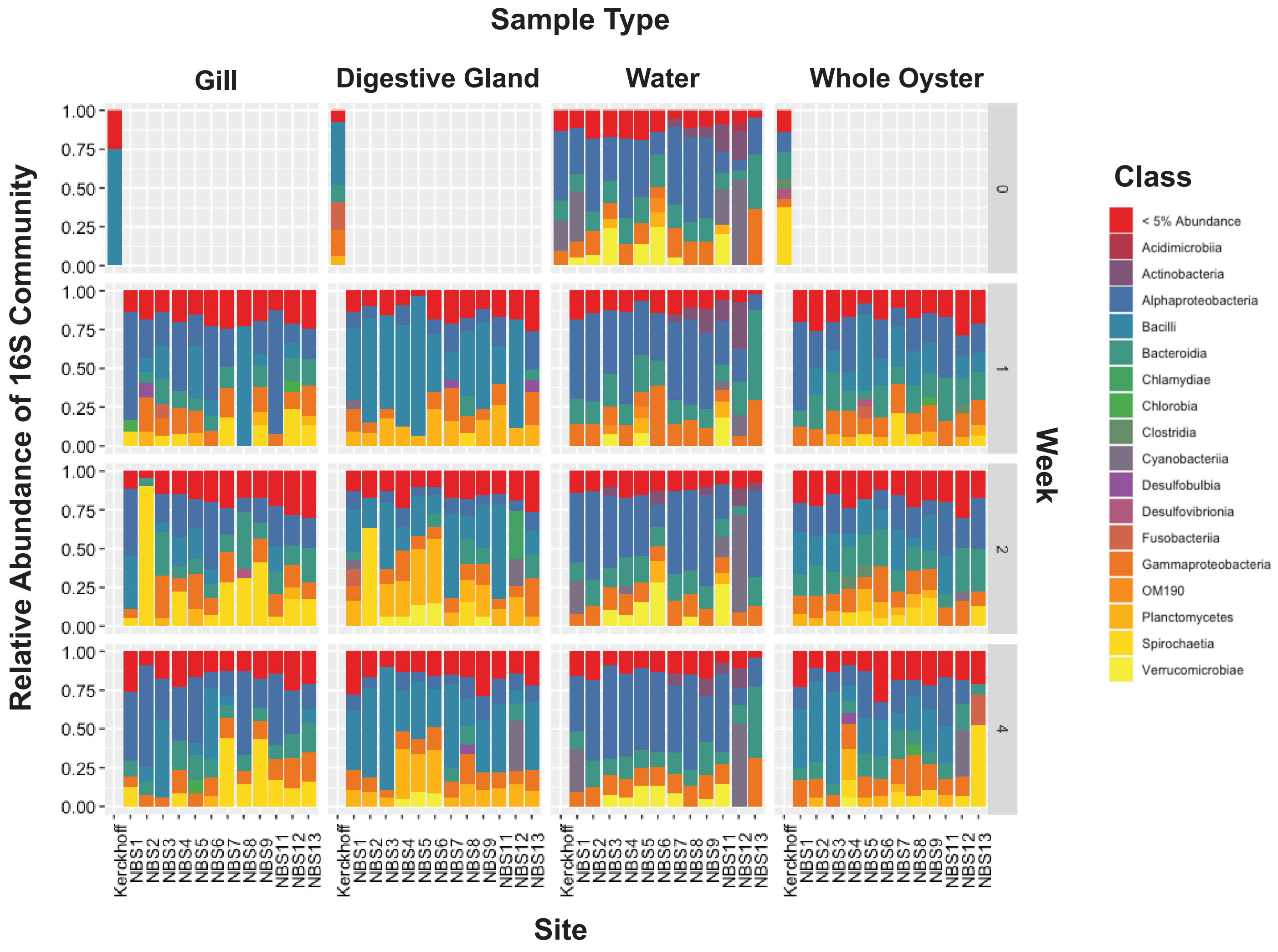

Fig S2: Taxonomic composition of oyster and water microbial communities over time. Relative abundance of 16S microbial communities for all sample types (water, whole oyster, gill, and digestive gland) at each week and site, including the Kerchoff facility following depuration (pre-deployment). Taxa are presented by class and classes representing less than 5% of the sequences in a particular sample type are grouped together (depicted by the red <5% category). Week 0 for oyster samples indicates the composite sample collected immediately following depuration, and Week 0 for water indicates water samples collected at each site at the time of oyster deployment.

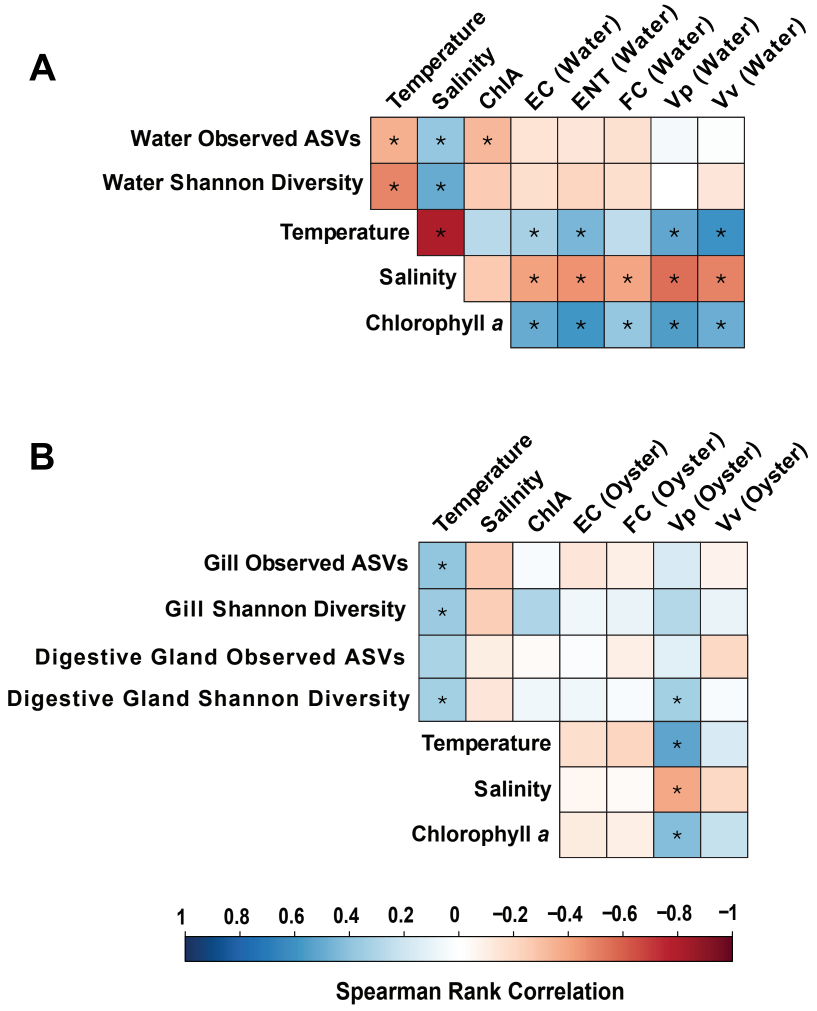

Fig S3: Spearman rank correlations of alpha diversity metrics, environmental variables, and target bacteria concentrations. Observed ASVs and Shannon Diversity alpha diversity metric correlations are shown for (A) water samples and (B) oyster tissue samples. Blue squares indicate positive correlations while red squares indicate negative correlations between variables.

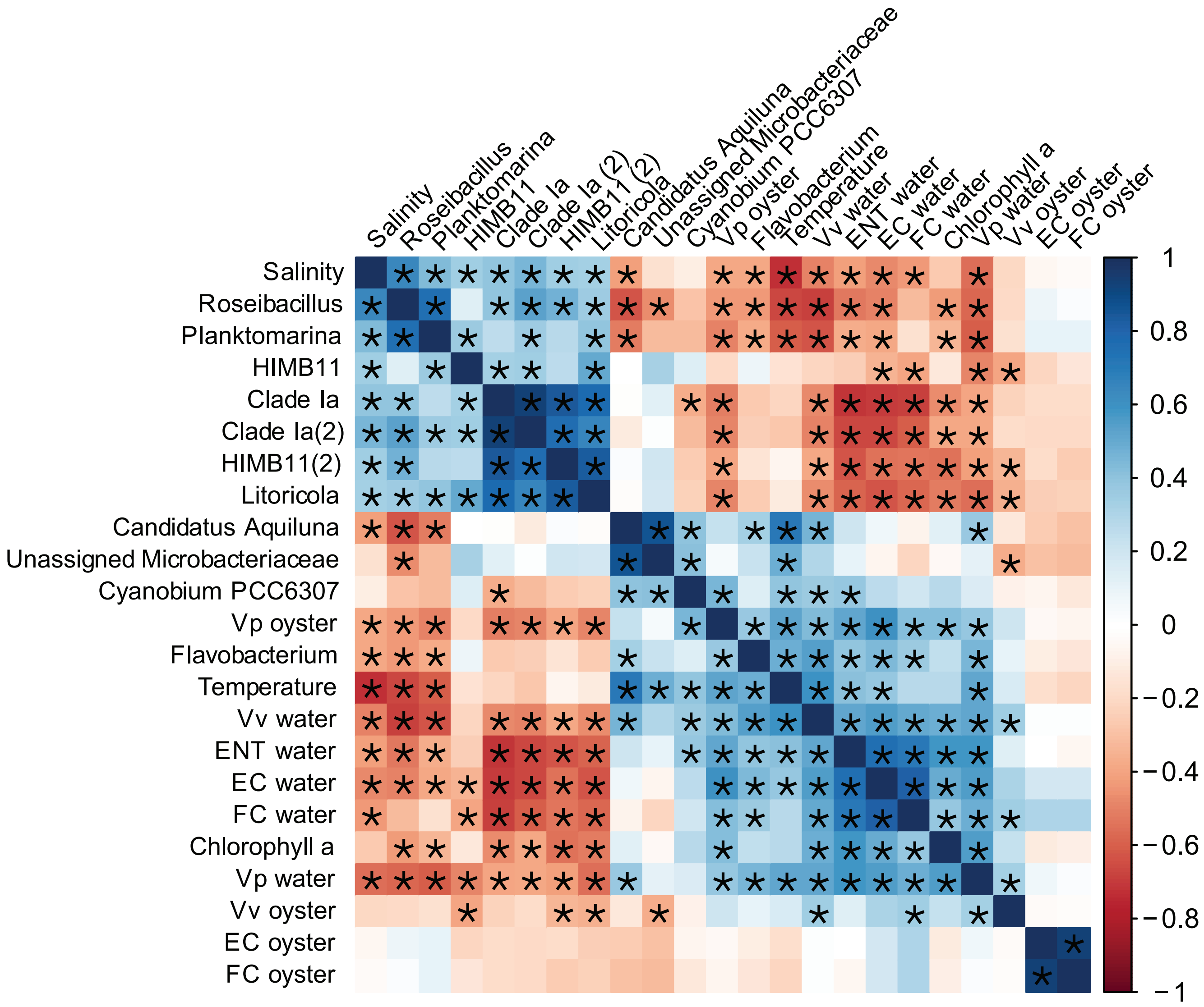

Fig S4: Spearman Rank correlations between fecal and vibrio bacteria concentrations, environmental variables, and the top 10 most abundant ASVs and specific bacteria of interest in <u>water</u> samples. Environmental variables (temperature, salinity, and chlorophyll *a*), and target bacteria concentrations are quantitative values, while microbiome taxa of interest are relative abundance. Bacterial taxa are annotated to the best available taxonomic resolution and are further described in Additional File 6. Numbers in parentheses indicate multiple ASVs with the same annotation. Target bacteria abbreviations for water and oysters are: EC = *Escherichia coli*, FC = fecal coliform bacteria, Vp = *Vibrio parahaemolyticus* and Vv = Vibrio vulnificus. * denotes a significant correlation (p-value = <0.05), and blue squares indicate a positive correlation while red squares indicate a negative correlation.

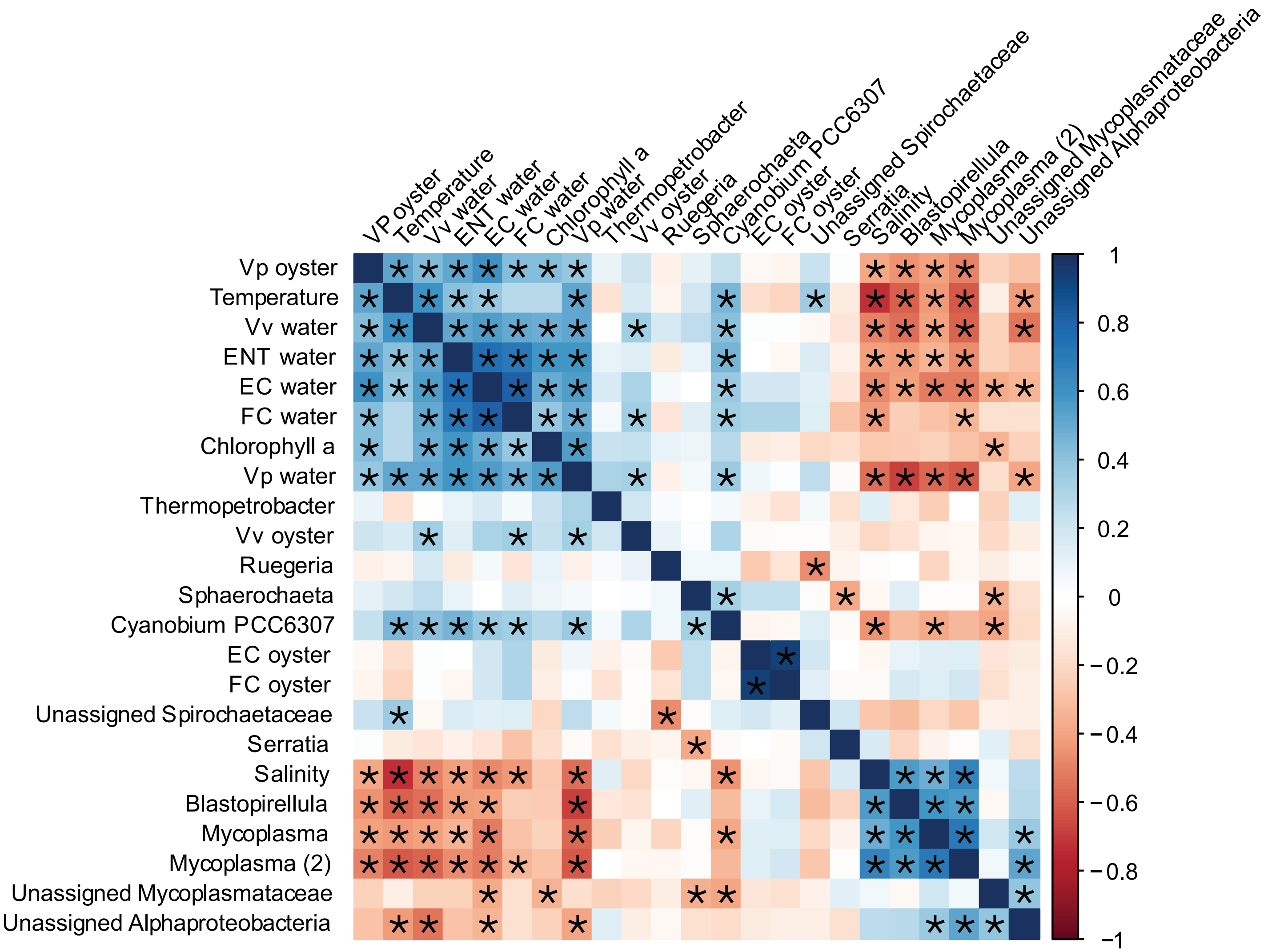

Fig S5: Spearman Rank correlations between fecal and vibrio bacteria concentrations, environmental variables, and the top 10 most abundant ASVs and specific bacteria of interest in whole oyster samples. Environmental variables (temperature, salinity, and chlorophyll *a*), and target bacteria concentrations are quantitative values, while microbiome taxa of interest are relative abundance. Bacterial taxa are annotated to the best available taxonomic resolution and are further described in Additional File 6. Numbers in parentheses indicate multiple ASVs with the same annotation. Target bacteria abbreviations for water and oysters are: EC = *Escherichia coli*, FC = fecal coliform bacteria, Vp = *Vibrio parahaemolyticus* and Vv = Vibrio vulnificus. * denotes a significant correlation (p-value = <0.05), and blue squares indicate a positive correlation while red squares indicate a negative correlation.

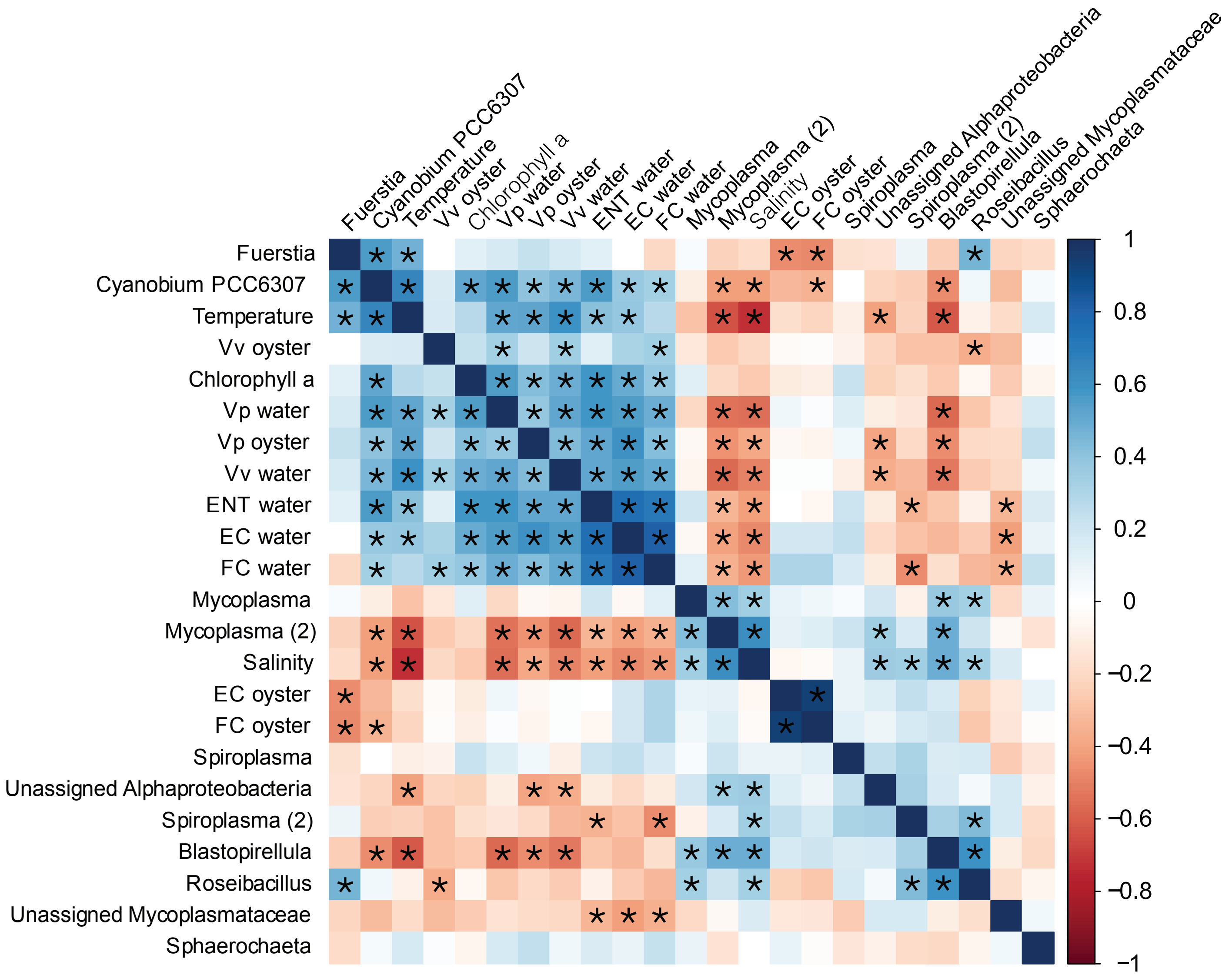

Fig S6: Spearman Rank correlations between fecal and vibrio bacteria concentrations, environmental variables, and the top 10 most abundant ASVs and specific bacteria of interest in digestive gland samples. Environmental variables (temperature, salinity, and chlorophyll *a*), and target bacteria concentrations are quantitative values, while microbiome taxa of interest are relative abundance. Bacterial taxa are annotated to the best available taxonomic resolution and are further described in Additional File 6. Numbers in parentheses indicate multiple ASVs with the same annotation. Target bacteria abbreviations for water and oysters are: EC = *Escherichia coli*, FC = fecal coliform bacteria, Vp = *Vibrio parahaemolyticus* and Vv = Vibrio vulnificus. * denotes a significant correlation (p-value = <0.05), and blue squares indicate a positive correlation while red squares indicate a negative correlation.

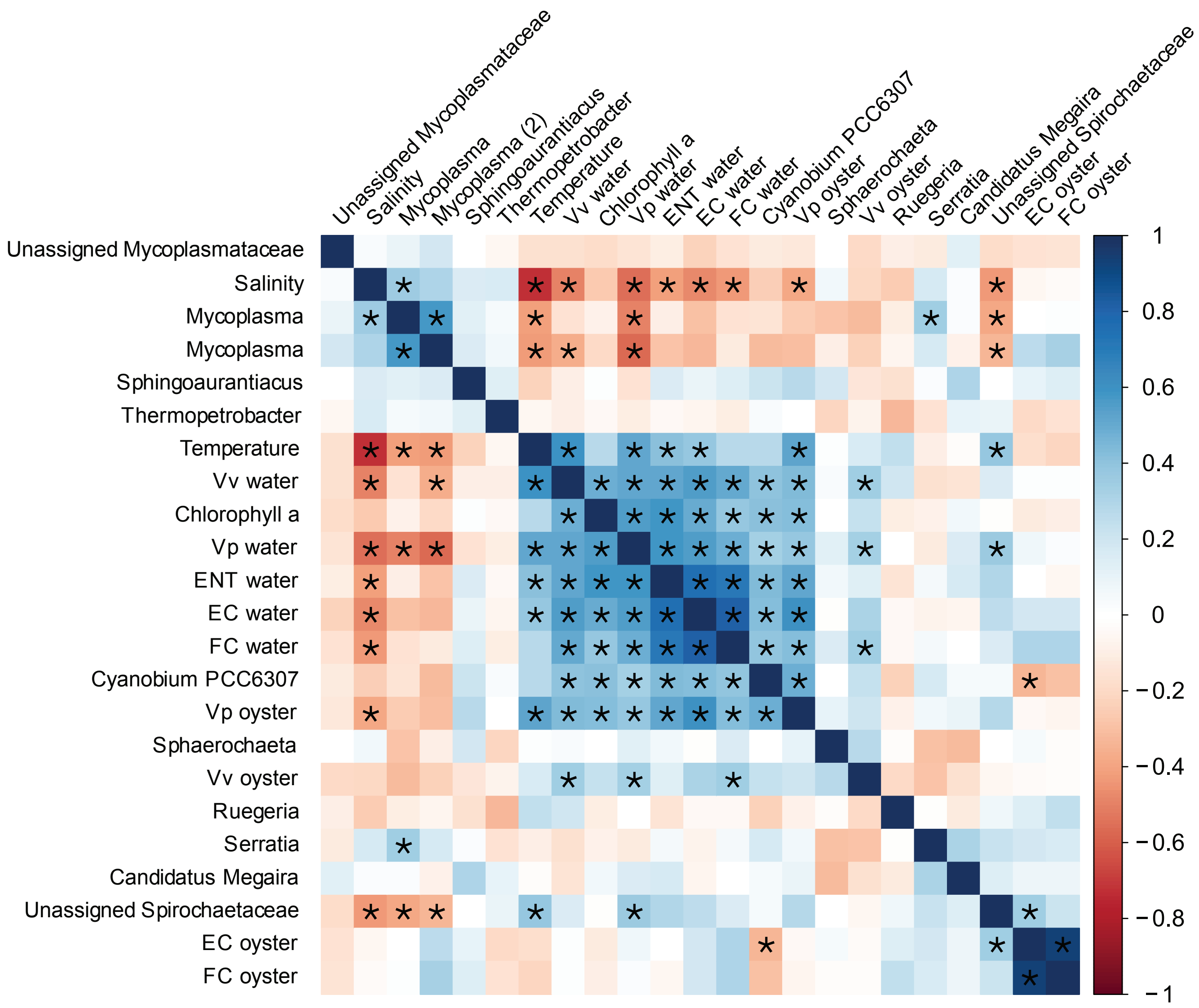

Fig S7: Spearman Rank correlations between fecal and vibrio bacteria concentrations, environmental variables, and the top 10 most abundant ASVs and specific bacteria of interest in gill samples. Environmental variables (temperature, salinity, and chlorophyll *a*), and target bacteria concentrations are quantitative values, while microbiome taxa of interest are relative abundance. Bacterial taxa are annotated to the best available taxonomic resolution and are further described in Additional File 6. Numbers in parentheses indicate multiple ASVs with the same annotation. Target bacteria abbreviations for water and oysters are: EC = *Escherichia coli*, FC = fecal coliform bacteria, Vp = *Vibrio parahaemolyticus* and Vv = Vibrio vulnificus. * denotes a significant correlation (p-value = <0.05), and blue squares indicate a positive correlation while red squares indicate a negative correlation.
